# Cooperative breeding does not mitigate declines in offspring care with cool and wet conditions in a temperate Australian songbird

**DOI:** 10.1098/rsos.250020

**Published:** 2025-03-05

**Authors:** Jenna N. Diehl, Abigail H. Robinson, Gregory T. Taylor, Rhiannon Myhre, Anne Peters

**Affiliations:** ^1^ School of Biological Sciences, Monash University, Clayton, Victoria 3800, Australia

**Keywords:** climate change, cooperation, iButton, micro-thermologger, nest temperature, parental care

## Abstract

Adverse climatic conditions can decrease reproductive success by reducing parents’ ability to provide enough resources to growing young. Here, we address the hypothesis that helpers at the nest can buffer the negative effects of challenging climatic (cool and wet) conditions in cooperatively breeding superb fairy-wrens. We first established that public records are adequate to quantify climate effects: temperatures recorded at a nearby meteorological station explained total offspring care equally well as microclimate temperatures measured inside the nest and near the nest site. We then compared the effects of temperature and rainfall on offspring care in pairs with and without helpers and found that (i) at lower, more energetically challenging temperatures, nestlings receive larger prey and more prey biomass, and females brood young nestlings more, but these increases in care occur regardless of helper presence; (ii) groups with helpers provide more prey biomass during dry conditions, but higher rainfall in the previous week reduces this to the level of unassisted pairs. Overall, cooperative breeding in superb fairy-wrens does not appear to buffer challenging (cool and wet) conditions: helpers do not mitigate the effects of cool temperatures and although groups with helpers deliver more food, this benefit disappears during periods with high rainfall.

## 1. Introduction

Understanding the effects of climatic conditions on animal populations is crucial for understanding the threat of climate change for biodiversity around the world [1]. Climatic conditions are expected to become more extreme and more variable [2], and these changing conditions challenge biodiversity worldwide, including birds [3]. For many species, the young are especially sensitive to environmental conditions and for altricial birds in particular, the maintenance of body temperature can be difficult when nestlings lack feathers for insulation and are still developing endothermy [4]. At low ambient temperatures, their energy costs increase to promote heat production and at high temperatures, both energy and water costs increase to promote heat dissipation [5]. Expending energy on thermoregulation can trade off with energy available to maintain rapid growth [6,7] and even short periods of adverse climatic conditions during early life can result in reduced growth [8–11] and life-long impacts [12,13] such as impaired adult survival, reproduction and overall fitness [14–16]. Therefore, it is important that the young receive additional nutrition during cold or hot conditions to compensate for their costs of thermoregulation. However, these conditions may also affect parents’ needs, and since they must balance current nestling needs with self-maintenance and future reproduction, if these parental needs prevail, young may instead receive reduced care during challenging climatic conditions [17,18].

Cooperative breeding has been proposed to buffer effects of unfavourable climatic conditions for parents and nestlings [19]. Cooperative breeding is a social strategy where parents are supported by additional group members, or ‘helpers’, that provide additional care to young that are not their own [20]. Such help can be compensatory, allowing breeders to keep provisioning static or reduce it [21,22] and/or additive, with increased investment from helpers increasing overall food brought to young [23,24]. Consequently, during unfavourable conditions, the additional support from helpers may mitigate the workload of parents while ensuring nestlings’ needs are still met. These contributions to offspring care, combined with the fact that cooperatively breeding species are disproportionately common in harsh or unpredictable environments [25,26] (but see [27,28]), suggest that a benefit of cooperative breeding may be buffering of environmental effects on productivity. For example, in white-browed sparrow-weavers (*Plocepasser mahali*), the presence of female helpers reduces rainfall-associated variance in reproductive success [29]. The main proposed mechanism by which such reproductive buffering would come about is an investment by helpers in offspring care. This environmental buffering hypothesis proposes that the contribution of helpers to offspring care varies with environmental conditions and becomes relatively more important during more challenging conditions [30–32].

Several field studies of cooperatively breeding birds have tested the hypothesis that the relative contribution to offspring care by helpers increases during unfavourable climatic conditions, with mixed results. In support of the hypothesis, one previous study in southern pied babblers (*Turdoides bicolor*) found that the provisioning rate in helpers decreased less steeply with temperature than in dominants [33]. However, a later study showed the decline in provisioning rate is offset by increases in prey size, and total biomass delivered by dominants does not change with temperature [34]. Moreover, relative helper contributions to incubation decline under more challenging climatic conditions, opposite to the pattern predicted by the buffering hypothesis [32]. Similarly, in colonial cooperatively breeding sociable weavers (*Philetairus socius*), there was a positive effect of rainfall on the condition of young fledged in pairs but not for groups, which might suggest a positive effect of helpers during dry conditions. However, this effect was very small and there was no evidence for greater offspring care by helpers during low rainfall [35]. Several other cooperatively breeding birds similarly do not show a change in helper contributions across climatic conditions [30,31,36] or show a decline in the relative importance of care by helpers under more challenging climatic conditions [37]. Overall, there thus appears to be limited support for the environmental buffering hypothesis.

However, all these studies only considered increasing temperature (hot conditions) and most focused on provisioning rate (except [34]). Undetected increases in size or quality of prey delivered to offspring could conceal or reveal changes in relative helper contribution [32,38]. Additionally, helpers may compensate for other types of nestling care. For example, in many pair-living species, when only the female broods the young, provisioning rates decrease when females spend more time brooding, e.g. in cool conditions (*Dumetella carolinensis* [39]; *Chlorocharis emiliae* [40]); increased investment in offspring care by helpers could compensate for reductions in provisioning by the female due to her needing to spend more time brooding, but this appears not to have been considered in cooperative breeders.

A potentially important, but overlooked, source of variation in climate effects on offspring care is that temperature varies spatially within an environment. All previous studies exploring the effects of climatic conditions on provisioning have used either air temperatures from a weather station on-site or a nearby meteorological station. Habitats comprise many thermal environments [41]; local temperature may differ from air temperature [42] and nest temperature may differ from air temperature, but also from local microhabitat temperature [43,44]. These differences are important to consider, as the temperature affecting the nestlings, which determines their thermoregulatory costs, may be different from the temperature experienced by the adults outside of the nest. Broadly available temperature data from weather stations may therefore not accurately reflect the thermal environment that determines the needs of the nestlings or the experiences of the adults and may explain, in part, the lack of evidence for a buffering effect of helpers on rates of offspring care during harsh environmental conditions.

Here, we determine how climatic conditions affect cooperative offspring care in free-living superb fairy-wrens (*Malurus cyaneus*), a small insectivorous songbird of southeastern Australia. Superb fairy-wrens are facultative cooperative breeders: subordinate helpers may assist a dominant breeding pair with offspring provisioning [45] while only the female incubates and broods the young. The climate in our study area is temperate, with relatively cold and wet winters and warm and dry summers. For our study species, challenging climatic conditions are represented by temperatures below 20–25°C or above 30–35°C, as these increase energy and water requirements for adults and nestlings, respectively [5,46]. Additionally, rain may exacerbate the challenge of cold conditions by increasing cooling or mitigate the challenge of warm conditions by enabling cooling. Finally, cold or hot temperatures, or periods of high rainfall may affect the activity and the availability of invertebrates [47–49], and thereby limit foraging opportunities or success or the quality of prey (size, water content) provided.

We quantify nestling care by breeders and helpers and climatic conditions experienced by adults and nestlings in natural nests to determine (i) the temperature cues that best explain adult behaviour, directly comparing those that the nestlings experience (in-nest) and those the adults experience, either locally (at the nest site) or broadly (derived form an on-site weather station or a nearby Bureau of Meteorology station); (ii) the effect of temperature and rain variation on offspring care. We include multiple aspects of care—provisioning rate, prey size and water content, total biomass delivered and nestling brooding—not only to more accurately assess relative contributions of breeders and helpers, but also to identify how any potential changes in total amount of care provided come about. Specifically, we test the predictions that (i) in-nest temperatures will best explain adult behaviours if offspring care is directly dictated by the needs of the nestlings; (ii) offspring care will change with temperature nonlinearly, with increased care provided at the coldest and hottest nest temperatures; and (iii) this increased provisioning will be brought about by helpers increasing their (relative) amount of care at these temperatures.

## 2. Methods

### 2.1. Study species

Superb fairy-wrens are a small (average mass: 9 g) passerine found in southeastern Australia. The wrens are year-round resident, facultative cooperative breeders [45], and during the breeding season (September to February) they form stable breeding pairs or groups and actively defend strict breeding territory boundaries [50]. Groups consist of a dominant breeding pair and 0−4 subordinate helpers which are typically male [51]. Helpers are typically offspring from previous breeding seasons that delayed dispersal, but helpers are often unrelated to the brood due to high levels of extra-pair mating [45]. The dominant breeding female will build the nest, incubate eggs and brood nestlings, but all members of the group will defend the nest and territory and feed nestlings. The dominant female typically provisions at the highest rate [20,52–54] and contribution from helpers can be additive, increasing the total provisioning to the nest [52,55,56]. However, in some studies with superb fairy-wrens, helper contribution was compensatory and the total provisioning rate was unaffected by helpers because the dominant males reduced the provisioning rate when helpers were present in the group [53,57,58]. This variability may partly be explained because none of the previous studies included the effects of climatic conditions, which may affect the decision for offspring care (i.e. prioritizing the young or prioritizing self-maintenance).

Sensitivity to temperature of nestlings and adults differs: the thermoneutral zone, or the range of temperatures where metabolic rate (measured by flow-through respirometry; see [5] for details) and energy costs are minimal, is between 25 and 35°C in adults [46] and between 33 and 42°C in nestlings [5]. Energy costs increase below the thermoneutral zone in order to promote heat production and increase above the thermoneutral zone to promote heat dissipation. In nestlings, temperatures above 34°C are also associated with linearly increasing water loss [5] but water loss in adults was not measured [46]. These thermal profiles were measured in very dry, high-airflow respirometry chambers and in natural conditions with higher humidity, thermal tolerance may be reduced [59]. For our study species, challenging thermal conditions are thus represented by temperatures below 20–25°C or above 30–35°C, as these presumably increase energy and water requirements for adults and nestlings.

### 2.2. Study site and field methods

We studied superb fairy-wrens in southeastern Australia, at Lysterfield Park, Victoria (−37° 56′ 56.40″ S, 145° 17′ 45.60″ E), on the traditional land of Bunurong Boon Wurrung and Wurundjeri Woi Wurrung peoples of the Eastern Kulin Nation. The park consists of open woodland, with areas of dense shrubs and open grassland and the climate is temperate, with typically cool/wet winters and warm/dry summers. With climate change, warmer mean temperatures, more heatwaves and less rainfall falling in fewer, heavier falls are forecasted (https://reg.bom.gov.au/state-of-the-climate/).

Fieldwork was conducted over two breeding seasons (September to February 2020/2021, 2021/2022); the average daily maximum temperature was 22.4°C (±4.1 s.d.), the average daily minimum temperature was 11.0°C (± 2.6 s.d.) and average monthly rainfall was 71 mm (±41 s.d.) (Bureau of Meteorology Scoresby station 86104). All research was approved by Monash University School of Biological Sciences Animal Ethics Committee (16348, 29007, BSCI/2018/01), Department of Environment Land Water and Planning and Parks Victoria (10010211, 10009936, 10008704), Parks Victoria access agreement (AA-0000004), and Australian Bird and Bat Banding Scheme (2230, 3641).

All individuals were banded with a unique combination of one numbered metal leg band, one coloured metal leg band and two coloured plastic leg bands. We determined breeding group size and social status of group members (dominant or helper) by observations of dominance (displacement, etc.) and breeding behaviours. In our population, 48% of pairs had at least one helper of either sex (mean 1.4 ± 0.6 s.d.), typically the philopatric young of the dominant female. We observed nest-building females to locate nests, which were checked every 3 days (avg 2.7 days ± 1.8 s.d. days) and monitored for egg laying, hatching and fledging, with generally 3−4 eggs laid (3.0 ± 0.5 s.d.). For nests not found during the building stage, observations of female incubation behaviour or of any adult carrying food were used to locate nests. Because nests were checked every 3 days, hatching was rarely observed; therefore all nestlings were aged based on their developmental characteristics (i.e. primary and body feather development)—from daily observations of nestling development for a number of nests, we know that developmental stage accurately reflects the number of days since hatching (hatch day = 1).

### 2.3. Offspring care data collection

To record activity at the nest, micro-cameras (CopCam HD Wireless Security Camera, Atomic Beam) were placed at 64 nests when nestlings were day 6 and/or day 8 (19 nests at both ages). The cameras (2.5 × 2.5 × 2.5 cm^3^) were painted dark green/brown for camouflage and placed 5−15 cm from the nest entrance, attached to a small stake or nearby vegetation, between 07.00 and 14.00. The cameras were motion triggered and recorded for 1−3 min at a time, covering on average 4.7 ± 1.6 (s.d.) hours in total. To verify the performance of the cameras, at 25 nests, simultaneous 1 h nest watches were conducted by observers with binoculars: cameras captured 92% of all nest visits (100% = total nest visits recorded by both methods combined), while in-person nest watches captured 57% of the total (unpublished data). The first hour of footage collected after camera placement was not included in data collection to avoid any potential effect of disturbance due to camera placement. For all nest visits, we recorded: the identity of the bird arriving at the nest, time of arrival, duration at nest and whether the nestlings were fed and/or brooded. For three nests, camera placement on day 6 did not allow identification of the group members and per-individual provisioning rates could not be obtained. Data were summed for each hour of the day (to allow alignment with temperature data). If <30 min of observation was available for the first or last hour of the recording period, that hour was removed from the data set. If ≥30 min of data were available, provisioning rates were scaled to a full hour and rounded to the nearest integer.

Superb fairy-wrens are generalist insectivore single prey loaders that forage or provision one item at a time [60], allowing us to estimate the size of the food item (56% of observations) and the type of prey (26% of all observations). Whenever a bird’s beak was visible in the camera frame, prey length was estimated relative to the beak (e.g. 1 = same length as the adult beak, 0.5 = half the length); if the prey item was too small to be visible in the beak, but the beak was fully visible, it was scored as 0.1, with prey type of micro (see electronic supplementary material, table S1). Prey size was converted to centimetres given an average adult superb fairy-wren bill length of 0.78 cm for our population (unpublished data). Total prey biomass (cm h^−1^) delivered per hour was calculated as average prey size multiplied by the provisioning rate. Prey were grouped into orders and % water was assigned based on published values (for details, see electronic supplementary material, table S1). Prey % water was multiplied by size and averaged per hour to derive an estimate of average water content of prey delivered (%). For the analyses of prey characteristics (size, biomass and water content), only nests with at least 50% of prey identified were used. Pearson’s correlations between average prey size and average water content and between provisioning rate and total biomass were relatively high (*r* = 0.71 and 0.63, respectively); all other correlations were <0.4 (electronic supplementary material, table S2a).

### 2.4. Climate data collection

Hourly temperature and daily rainfall data were obtained from the Bureau of Meteorology’s (BoM) Scoresby station (86104) 9 km away from the field site. Cameras were never placed during rain; rain data were summarized as the sum of the total rainfall from the preceding 7 days. Hourly local temperatures were collected by an on-site weather station (Wireless Vantage Pro2, Davis Instruments) placed in a central location of the field site.

In-nest temperatures were collected using small (17 mm diameter) temperature sensors (micro-thermometers; Thermochron DS1921G-F5, Maxim Integrated) placed in brown/green thin cotton bags and attached to the interior roof of the nest using green PVC garden wire. Placement in the roof of the nest ensured that the temperature sensor was not in contact with the eggs, nestlings or incubating/brooding females, to obtain nest temperature estimates largely independent of the presence of the female or nestlings in the nest. The inside of the nest was small with little variation between nests (average distance from roof to bottom of nest was 7.93 cm ± 1.28 s.d.) and brood size was small (1–4 nestlings; average 2.72 ± 0.73 s.d.), thus we expect the difference between temperature measured in the roof and the temperature in the bottom of the nest (determining the nestling experience) to be relatively small.

Nest site temperatures were collected by a temperature sensor suspended in a plastic fob (DS9093A ibutton Fob, Maxim Integrated) in vegetation about 5−10 cm under the nest; sensors were never exposed to direct sunlight. These temperatures represent the immediate area around the nest, and most closely reflect the temperatures experienced by the adults (as superb fairy-wrens exclusively occupy their relatively small territory).

In-nest and nest site temperatures were recorded every 10 min and averaged per hour.

### 2.5. Statistical analysis

All analyses were conducted in R (version 4.3.1 [61]) . For each aspect of offspring care: (i) provisioning rates (feeds per hour), (ii) average prey size (cm), (iii) average water content (%), (iv) total biomass (cm h^−1^), (v) probability of the female initiating brooding and (vi) time spent brooding (min), we first aimed to determine the temperature cues that best explained adult behaviour (aim 1). The temperature sources were highly correlated and could not be included in a single model (all correlations >0.74; electronic supplementary material, table S2b); thus, we constructed four separate models and used Akaike information criterion (AIC) to compare model fit (‘aictab’ in *AICcmodavg* [62]) between the temperature sources (in-nest, microsite, on-site or BoM). Models with ∆AIC < 2 were considered to have a similar fit. The data source for these model comparisons only included nests for which all four temperatures were available (see electronic supplementary material, tables S3–S8). Subsequently, to determine how temperature and rain affected offspring care (aim 2), we constructed separate models for each aspect of offspring care (i)−(vi) using the full data set for the temperature source identified as providing the best model fit.

All comparison and full models included the following fixed effects: temperature (°C), total rainfall (mm) during the previous week, whether or not helpers were present (yes/no), and brood size (continuous, 1−4; accounting for the number of nestlings in the nest). The start time (time of the day the observation was started), hour of the day, date in the season (date first egg laid in the population = 1), and year were also included to account for temporal effects (i.e. higher feeding rates earlier in the day (e.g. [63]); reduced nest investment later in the season (e.g. [64,65])). Nestling age was also included in the models, and since day 6 nestlings likely have poorer thermoregulatory ability than day 8 nestlings, we tested the interaction between nestling age and temperature in full models. All continuous effects were mean-centred to aid in model interpretation and convergence. All models also included a temporal autocorrelation correction for each hour on each day (‘glmmTMB’, in package glmmTMB [66]), and random intercepts of Nest and Female identity to control for replication across nests and females. The group membership does not change with breeding seasons, and rarely between breeding season and thus Female identity can accurately capture group identity in our data set.

For models of (i) provisioning rates, we used a generalized linear mixed model (GLMM) with zero-inflated negative binomial distribution and a log-link function; zero-inflation was assumed to be constant across the data set and the negative binomial distribution accounted for overdispersion. Energy costs increase at low and high temperatures; thus, we tested for nonlinear effects of temperature on provisioning rate by including its quadratic. For the models of (ii) average prey size and (iii) average water content, we used GLMMs with Gaussian distribution and the log-link function and for (iv) total biomass, we used a negative binomial distribution and log-link function to account for overdispersion. For (v), the probability of the female initiating brooding (yes/no per hour), we used GLMMs with binomial distribution and *logit* link function. For (vi), total time spent brooding, we only included hours where brooding was observed and used gamma distribution and *log* link function.

To estimate whether helpers could mitigate adverse conditions (aim 3), we tested in full models (i)–(vi) interactions between helper presence (yes/no) and rain, and helper presence (yes/no) and temperature. If a significant interaction with helper presence was identified, because continuous variables were mean-centred, we derived estimate ± s.e. and *p*-value for unassisted pairs (the default reference category) from the main effect for the climate variable and then switched the reference category to groups (helpers present) to derive the alternative estimate and *p*‐value. Additionally, we investigated whether helpers responded differently to temperature and rainfall by constructing models of per-individual (vii) provisioning rates (feeds per hour) and (viii) average prey size (cm). Models were the same as described above for provisioning rate and average prey size per nest, with additional fixed effects of sex (male/female), social status (dominant/helper), the interactions of social status and temperature or rain and the random effect of bird identity. We also included the interactions between sex and social status and sex and helper presence, since male and female superb fairy-wrens are known to moderate parental care differently [52]. When significant interactions with climate variables were present, we computed the overall effect and statistical details for climate parameters by applying sum-to-zero contrasts on the factor involved in the interaction.

After model fitting, the residuals were checked for normality, zero-inflation, and overdispersion (package *DHARMa* [67]) and to ensure that there was no evidence for temporal autocorrelation in the model residuals (‘acf’, in package *stats* [61]). Non-significant interactions were removed and reintroduced into the final model one by one to derive the statistical details presented in electronic supplementary material, tables S10−S12. Sample sizes for all analyses can be found in electronic supplementary material, tables S3−S12. Model predictions and 95% confidence intervals were calculated using ggpredict (package *ggeffects* [68]), accounting for all random effects in the final model and variables not of interest included at their mean value. We present model-predicted values ± s.e. unless indicated otherwise.

## 3. Results

### 3.1. Comparison of the four temperature sources

Temperatures recorded in the nest, and to a lesser extent at the nest site, were warmer (by on average 4.32°C ± 3.33 s.d. and 1.70°C ± 3.08 s.d., respectively) and more strongly correlated to each other than to the temperatures recorded by the weather stations (that were also highly correlated to each other; details in electronic supplementary material, table S2). However, when comparing how well the different sources of temperature data predicted adult responses, we found there were no differences in model fit (all ∆AIC < 1.40) between the models of provisioning rate (electronic supplementary material, table S3), total biomass delivered to the nest (electronic supplementary material, table S6), and time the female spent brooding the nestlings (electronic supplementary material, table S8). For probability of initiating brooding, the models using in-nest, nest site or BoM temperature all fitted equally well (all ∆AIC < 1.22; electronic supplementary material, table S7) but using on-site temperature had a worse fit (∆AIC = 4.60). Similarly, for the models of average water content of prey, using nest site, on-site, or BoM temperatures all fitted equally well (all ∆AIC < 1.96; electronic supplementary material, table S5) but using in-nest temperature had a worse fit (∆AIC = 2.49). Therefore, for all full models for these dependent variables, we used BoM temperature, for which we had the largest temperature data set.

For average prey size, temperature recorded by BoM provided a less good model fit than nest site temperature (on-site weather station and in-nest temperature were similar to BoM with 3.22 < ∆AIC < 3.61 compared to nest site temperature; electronic supplementary material, table S4). However, because full models with BoM and nest site temperature produced similar results (electronic supplementary material, table S9), for consistency and because it allowed for larger sample size, final models presented for average prey size also use BoM temperature data.

### 3.2. Effects of climate variation on offspring care

**Figure 1.**
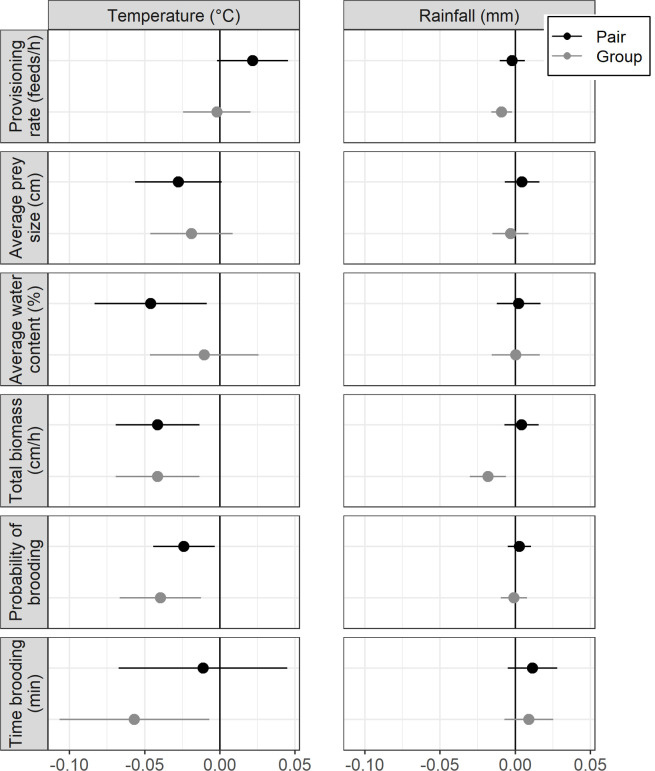
The effect of temperature and rainfall on offspring care is similar in pairs and groups with helpers, except that increased rainfall results in a decrease in total biomass in groups but not in pairs. For pairs without helpers (black) and groups with helpers (grey), the figure shows the effect size and s.e. of air temperature (°C, hourly) and rainfall (mm, during the preceding week) on each aspect of offspring care: provisioning rates (feeds per hour), average prey size (cm), average water content (%), total biomass (cm h^−1^), probability of brooding and time brooding (min). Climate data were obtained from the nearest BoM station (86104; bom.gov.au).

Hourly rate of provisioning to the nest was not affected by temperature, linearly (figure 1; electronic supplementary material, figure S1a; 0.01 ± 0.01, *p* = 0.33) or nonlinearly (quadratic effect: *p* = 0.44; electronic supplementary material, table S10a). Total rainfall during the week preceding the observation had a significant negative effect on provisioning rate (figure 1; electronic supplementary material, figure S2a; −0.01 ± 0.00, *p* = 0.04). Hourly provisioning rate also decreased with date in the season and increased with brood size, but there was no significant effect of nestling age (electronic supplementary material, table S10a).

Average size of prey delivered to the nest decreased with increasing temperature (figure 1; electronic supplementary material, figure S1b; −0.02 ± 0.01, *p* = 0.03), but rainfall during the preceding week had no effect (figure 1; electronic supplementary material, figure S2b; *p* = 0.89). Older (day 8) nestlings and larger broods received larger prey (electronic supplementary material, table S10b). Average water content of prey tended to decline with increasing temperature, although not significantly (electronic supplementary material, figure S1c; −0.03 ± 0.02, *p* = 0.06) and water content did not change with rainfall during the preceding week (figure 1; *p* = 0.92). There was an increase in water content during the day and with increased brood size, but no significant effect of nestling age (electronic supplementary material, table S10c).

The overall effect of temperature on total biomass delivered to the nest was negative (−0.04 ± 0.01, *p* = 0.003). However, there was a significant interaction between nestling age and temperature, such that for older (day 8) nestlings, total biomass decreased significantly with increasing temperature (electronic supplementary material, figure S1d; −0.07 ± 0.02, *p* = 0.003) but this decline was not significant for younger (day 6) nestlings (−0.02 ± 0.01, *p* = 0.17). Increasing rainfall during the preceding week was associated with a decline in biomass delivered to the nestlings, but this was not significant (−0.01 ± 0.00, *p* = 0.10); there was a significant interaction with the presence of helpers, with a strong negative effect of rainfall on biomass delivered by groups (for details, see §3.3). There was an increase in biomass delivered with increased brood size but no overall effect of nestling age (electronic supplementary material, table S10d).

Probability of brooding declined significantly with increasing temperature (figure 1; electronic supplementary material, figure S3a; *p* < 0.001) as did time spent brooding, although the effect was not significant (electronic supplementary material, figure S3b; *p* = 0.07). There was no effect of rainfall on the probability of brooding (*p* = 0.79), or time spent brooding (*p* = 0.09). Older nestlings were brooded less often and less long (both *p* < 0.001; electronic supplementary material, table S11).

### 3.3. Effects of helpers on offspring care

**Figure 2.**
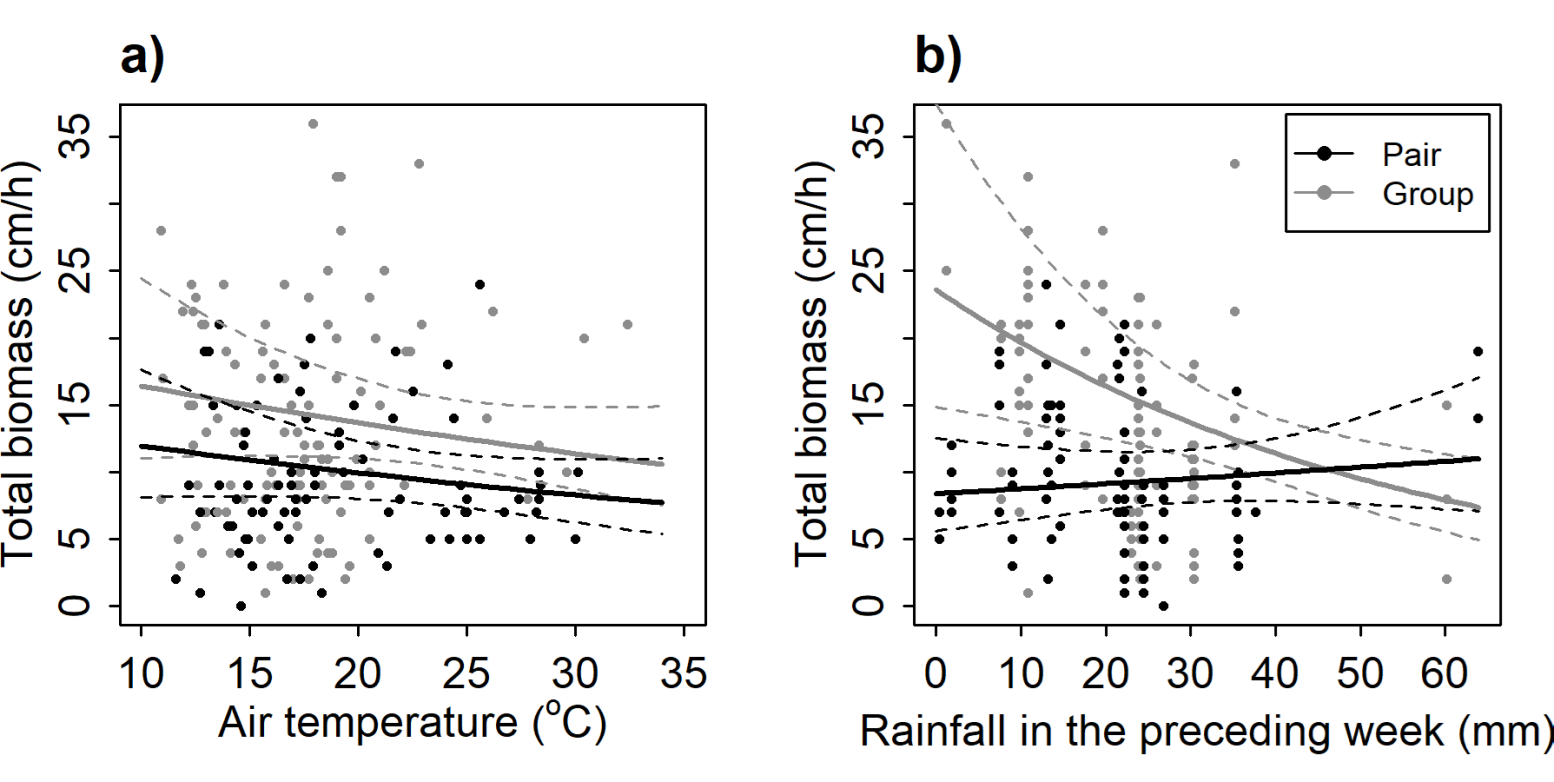
The response of pairs (black) and groups with helpers (grey) to climate conditions showing that, although helpers increase prey biomass, (*a*) this effect is not greater at lower daily maximum temperatures (no interaction between temperature and presence of helpers, *p* = 0.69; electronic supplementary material, table S10) and (*b*) increasing total rainfall during the preceding week resulted in a decline in total biomass in groups with helpers (*p* = 0.003) but not in pairs (*p* = 0.47). Thus, more challenging climatic conditions reduce the positive effect of helpers. Shown are average hourly air temperature (°C) and total rainfall from the preceding week as recorded by the BoM; dots are observed data; solid lines show the model predictions; and dashed lines the 95% confidence intervals.

Helper presence (yes/no) had no effect on provisioning rate (*p* = 0.46) and prey size (*p* = 0.67), but overall, groups delivered more biomass per hour than pairs (0.32 ± 0.11 cm h^−1^, *p* = 0.005; electronic supplementary material, table S10). Pairs delivered on average 9.60 cm h^−1^ (±0.11 s.e.) of biomass while groups delivered on average 13.19 cm h^−1^ (±0.10 s.e.) of biomass. However, increasing rainfall reduced the contribution of helpers to the total biomass delivered to the nest (figure 2; interaction *p* = 0.007): while total biomass delivered was higher in groups in dry conditions, total biomass declined with increasing rainfall in groups (−0.02 ± 0.01 cm h^−1^ mm^−1^, *p* = 0.003) while in pairs there was no effect of rainfall (0.004 ± 0.006 cm h^−1^ mm^−1^, *p* = 0.47; electronic supplementary material, table S10d). The response to temperature was similar in pairs and in groups (figure 1), and the presence of helpers did not modulate any other aspects of offspring care, with no effect on provisioning rates (*p* = 0.46), average prey size (*p* = 0.66) or water content of prey (*p* = 0.67), probability of brooding (*p* = 0.31) or time spent brooding (*p* = 0.91; electronic supplementary material, table S11).

All group members responded similarly to temperature and rainfall: there was no effect of social status (helper/dominant) on the effects of temperature or rainfall on per-individual provisioning rate or average prey size (electronic supplementary material, table S12; all interactions *p* > 0.19). All individuals decreased provisioning rate with increasing rainfall (−0.01 ± 0.004, *p* = 0.01) but rainfall did not affect average prey size (*p* = 0.54) and provisioning rate and average prey size were unaffected by temperature (all *p* > 0.25). There was a significant interaction between sex and status on provisioning rate: helper females provisioned less than dominant females, but helper males and dominant males provisioned at similar rates (electronic supplementary material, figure S4 and table S12a; *p* = 0.04). Accordingly, there was also an interaction between sex and the presence of helpers (*p* = 0.001): males provisioned less when helpers were present within the group, while females did not adjust feeding rate (0.10 ± 0.17 feeds per hour, *p* = 0.55). There was no effect of sex, status or their interaction on the average size of prey brought to the nest (electronic supplementary material, figure S4 and table S12b; all *p* > 0.12).

## 4. Discussion

This study investigated whether helper assistance with offspring care compensates for challenging climatic conditions in a temperate cooperatively breeding songbird. We found that helpers did not improve the effect of climatic conditions on offspring care. Although total biomass delivered to the brood increased as temperature decreased (and nestling needs increased), this increase in care was not due to helpers increasing their contribution. Additionally, provisioning rates in groups decreased as the previous week’s rainfall increased and although nestlings received more food in groups with helpers, this effect diminished as weekly rainfall increased. Overall, we thus found no evidence that helpers compensate for more challenging—cool or wet—conditions.

### 4.1. Temperature cues for parental care behaviours

Although temperatures experienced by adult birds differ from the thermal environment of their nestlings (electronic supplementary material, table S2), our results show that parental care responses are equally well explained by temperatures measured by micro-sensors, placed in or at the nest, by a weather station placed directly at our site, or a meteorological station 9 km away. To our knowledge, no studies previously explored how using different temperature sources may affect predictions of offspring care. Previous studies of potential buffering effects by helpers used temperatures collected by meteorological stations, away from the nest site [30,31,33–37], which may or may not represent the thermal experiences of nestlings or adults. The thermoregulatory needs of adults and nestlings may increase at different times, as the adults and nestlings are exposed to different temperatures, and this may make it difficult to understand and predict changes in adult behaviour. Despite the inherent validity of such concerns, and the differences in temperature response for nestlings and adults [5,46] our results showed that for the most part, these considerations were not important; for each aspect of offspring care, all temperature cues gave similar model fits and similar results. However, we did find that the average size of prey brought to the nest was best explained by temperatures collected from the nest site (best model fit) which may be because prey activity is affected more by local temperature near the nest or adults may have selected prey in response to temperature they directly experience. More detailed observations of prey activity or prey selection by birds would be required to investigate such potential mechanisms further. Nonetheless, despite these considerations, our results show that publicly available meteorological data, from nearby the study area, adequately explain effects of temperature on care provided to nestlings, which is reassuring since most studies rely on such data.

### 4.2. Negative effects of temperature and rainfall

At lower temperatures, average prey size was larger, and more total biomass was delivered to nestlings, although this effect was much smaller, and not significant, for younger (day 6) nestlings. Across the temperature range (10–33°C recorded by BoM; 11–42°C in the nest), the effect of temperature was linear, which agrees with our predictions based on nestling thermal sensitivity determined by respirometry [5]. Superb fairy-wren nestlings have a high thermoneutral zone, with the lower limit at 33°C and the upper limit at 42°C. For the range of temperatures they experienced in our study, even with nests being on average 4–5°C warmer than air temperatures recorded by BoM (electronic supplementary material, table S2), nestlings would have required less food as temperature increased. This agrees with a lack of evidence for nonlinear effects, as temperatures in our study did not exceed the upper limits, and thus nestlings likely did not require an increase in provisioning at higher temperatures. The larger and significant effect of temperature on provisioning for older (day 8) nestling may be due to older nestlings receiving larger prey or because they are brooded less by the female and therefore must expend more energy on thermoregulation, requiring more food, at lower temperatures.

Across the temperature range in our study (10–33°C recorded by BoM; 11–42°C in the nest), average water content of prey delivered to nestlings did not significantly change. Evaporative water loss for nestlings increases with increasing temperature (starting above 34°C when measured in a respirometry chamber [5]). We hypothesized that adults might compensate for water loss by delivering more prey or prey with higher water content as temperature increases. However, our results do not provide evidence of compensation for greater water need at higher temperatures. If anything, they show the opposite, as the average water content of prey tended to decrease, even if this was not significant (*p* = 0.06), the direction is opposite to the predicted effect of an increase. This may be because under conditions of natural humidity (as opposed to dry air of the respirometry chamber [5]), water loss does not increase, or not as rapidly, with increasing temperature. Average air temperature was relatively mild during the study period (<35°C; electronic supplementary material, table S2), only five nests recording nest temperatures >35°C, thus nestling water loss may have not increased enough to require compensation by parental provisioning. Nonetheless, a decline in prey water content with increasing temperatures is unlikely to be beneficial for nestlings. The decrease is more likely to be related to prey activity as the availability of the larger ground-dwelling prey with high water content (e.g. earthworms, insect larvae) decreases at higher temperatures [48,69,70].

Increased rainfall during the preceding week resulted in reduced provisioning rate, and a decline in prey biomass delivery in groups. We did not collect data on days when it was raining, as it is well known that provisioning and foraging decline or cease during rain [71–73]. The decrease in offspring care with increased rainfall during the week prior to the observation thus indicates a delayed effect of rain. Increased rainfall in preceding days may have limited the time available for the adults to forage, and adults may thus prioritize self-maintenance over provisioning of nestlings on the subsequent days when it is not raining [74]. During rainy periods and immediately after periods of rain, insects are also less active [47,49,75], which can make provisioning more difficult during rainy weeks, also resulting in reduced provisioning rates. An increase in periods of heavy rainfall, associated with climate change, may strain the ability of adults to provide for young in future climates.

### 4.3. Helpers do not buffer adverse conditions

Provisioning rate to the nest, the most commonly measured aspect of parental care, was not affected by helpers in the group. This lack of helper effect is consistent with some previous studies on superb fairy-wrens [53,57], which showed that the dominant males decreased provisioning rates when they had helpers, but not with other studies which showed that helper contribution was additive and increased the total provisioning rates [52,55,56]. Unfortunately, none of these previous studies measured prey size or type or incorporated climatic conditions, which is important as incorporating these factors might have explained why patterns in provisioning rate varied. For example, we found that the positive effect of helpers was only evident for the combined effect of provisioning and prey size—total biomass—and only during dry weeks.

Despite our more detailed estimates of offspring care, we found little evidence that the facultative cooperative breeding strategy of superb fairy-wrens buffers against challenging conditions. For all aspects of offspring care, the presence of helpers did not moderate effects of temperature or rainfall on the amount of care provided. First, the response to cooler, more challenging, temperature did not vary with presence of helpers: at lower temperatures, dominant females brooded young nestlings more while also maintaining higher feeding rates, with no increased assistance from helpers. Second, although greater total prey biomass was delivered to nestlings in groups with helpers, biomass delivered by groups decreased to the level of pairs with increasing rainfall. Thus, a positive effect of helpers on biomass was only evident during benign conditions (less rainfall during the previous week). Both results do not support the proposed buffering effect of cooperation, which predicts that the relative contribution of helpers increases during challenging conditions.

Our results are consistent with the majority of previous studies investigating whether cooperative breeding buffers challenging climatic conditions. These studies, all in a hot/dry climate, showed either no increase in relative contribution to offspring care by helpers [30,35–37] or a greater decline in provisioning by helpers during challenging—hotter and dryer—conditions [30,34,37] (but see [33] where the decline in provisioning on hot days was slightly less steep in helpers compared to breeders). These studies include superb starlings (*Lamprotornis superbus*) [76], where most effects of helpers on provisioning rates occur during benign conditions, even though this species belongs to the African starlings, the group of birds with arguably the strongest comparative evidence of harsher climate selecting for cooperation [26]. More individual field-based studies of climatic effects on helper contributions in a greater variety of cooperatively breeding species, from a greater variety of environments and climates, informed by temperature-dependence of energy needs of adults and offspring, are required to understand the selection processes behind the global association between cooperative breeding and climate [77,28,19].

## 5. Conclusions

Cooperative breeding has been suggested as a strategy to help buffer the costs of care during challenging climatic conditions. In our system, colder temperatures and wetter periods are likely more challenging, but helpers did not increase care in these conditions, confirming the majority of previous tests that failed to find support for the buffering hypothesis for offspring care (i.e. provisioning). However, few studies investigated multiple aspects of offspring care, and our results highlight how provisioning rate, prey size and type and brooding may vary independently; including multiple aspects of care might therefore alter conclusions, although doing so did not reveal a buffering effect in our study.

While previous studies showed that helpers do not mitigate impacts of hot and/or dry conditions, our results expand this result to cool and wet conditions, that also increase energy costs for nestling thermoregulation and likely hamper foraging success for adults. Implications for climate change likely do differ with temperature range, however, as increases in temperature associated with climate change may be beneficial in our population. Warming may reduce the thermoregulatory needs of nestlings, thereby lessening the offspring care load for adults and/or improving nestling condition (e.g. short heatwaves (*T*_max_ > 35°C) increase nestling telomere length in this species, indicative of better somatic maintenance [78]). However, if temperatures rise above the upper limit of the nestling thermoneutral zone, water and energy needs likely increase, and further research is needed to determine how offspring care may change during prolonged heatwaves.

## Data Availability

All data supporting the results are available in Figshare (https://doi.org/10.6084/m9.figshare.26068885.v1). Supplementary material is available online [79].
